# Discovery, Development, and Patent Trends on Molnupiravir: A Prospective Oral Treatment for COVID-19

**DOI:** 10.3390/molecules26195795

**Published:** 2021-09-24

**Authors:** Mohd. Imran, Mandeep Kumar Arora, Syed Mohammed Basheeruddin Asdaq, Shah Alam Khan, Saleh I. Alaqel, Mohammed Kanan Alshammari, Mohammed M. Alshehri, Ahmed Subeh Alshrari, Alreshidi Mateq Ali, Ahmed Muteb Al-shammeri, Bushra Dhuhayyan Alhazmi, Aishah Ali Harshan, Md. Tauquir Alam, Abida

**Affiliations:** 1 Department of Pharmaceutical Chemistry, Faculty of Pharmacy, Northern Border University, Rafha 91911, Saudi Arabia; saleh_alagel@hotmail.com (S.I.A.); tauquirpharm@gmail.com (M.T.A.); aqua_abkhan@yahoo.com (A.); 2 School of Pharmaceutical and Population Health Informatics, DIT University, Dehradun 248009, Uttarakhand, India; mandeepK.arora@dituniversity.edu.in; 3 Department of Pharmacy Practice, College of Pharmacy, AlMaarefa University, Dariyah, Riyadh 13713, Saudi Arabia; 4 College of Pharmacy, National University of Science and Technology, Muscat 130, Oman; shahalam@nu.edu.om; 5 Department of Pharmaceutical Care, Rafha Central Hospital, North Zone, Rafha 91911, Saudi Arabia; ii_kanan101@outlook.com; 6 Pharmaceutical Care Department, Ministry of National Guard-Health Affairs, Riyadh 11426, Saudi Arabia; M.Mansouralshehri@outlook.sa; 7 Medical Laboratory Technology Department, Faculty of Applied Medical Science, Northern Border University, Arar 91431, Saudi Arabia; ahmed.alsharari@nbu.edu.sa or; 8 Department of Medical Laboratory Sciences, Sulaiman ALrajhi University, Albukairah 51941, Saudi Arabia; ma.alreshidi@sr.edu.sa; 9 Pharmaceutical Affairs, Dammam Health Network, Eastern Health Cluster, Dammam 31146, Saudi Arabia; Shammeriam@gmail.com; 10 Faculty of Pharmacy, Northern Border University, Rafha 91911, Saudi Arabia; bushraalhaz96@gmail.com; 11 Department of Pharmaceutical Care, Northern Area Armed Forces Hospital, King Khalid Military City Hospital, Hafr Al-Batin 31991, Saudi Arabia; Ais7ah.har@gmail.com

**Keywords:** molnupiravir, EIDD-2801, MK-4482, EIDD-1931, patents, SARS-CoV-2, COVID-19

## Abstract

The COVID-19 pandemic needs no introduction at present. Only a few treatments are available for this disease, including remdesivir and favipiravir. Accordingly, the pharmaceutical industry is striving to develop new treatments for COVID-19. Molnupiravir, an orally active RdRp inhibitor, is in a phase 3 clinical trial against COVID-19. The objective of this review article is to enlighten the researchers working on COVID-19 about the discovery, recent developments, and patents related to molnupiravir. Molnupiravir was originally developed for the treatment of influenza at Emory University, USA. However, this drug has also demonstrated activity against a variety of viruses, including SARS-CoV-2. Now it is being jointly developed by Emory University, Ridgeback Biotherapeutics, and Merck to treat COVID-19. The published clinical data indicate a good safety profile, tolerability, and oral bioavailability of molnupiravir in humans. The patient-compliant oral dosage form of molnupiravir may hit the market in the first or second quarter of 2022. The patent data of molnupiravir revealed its granted compound patent and process-related patent applications. We also anticipate patent filing related to oral dosage forms, inhalers, and a combination of molnupiravir with marketed drugs like remdesivir, favipiravir, and baricitinib. The current pandemic demands a patient compliant, safe, tolerable, and orally effective COVID-19 treatment. The authors believe that molnupiravir meets these requirements and is a breakthrough COVID-19 treatment.

## 1. Introduction

COVID-19 is currently well known among the general population. This disease was first reported in Wuhan (China) in December 2019 and is caused by SARS-CoV-2 (2019-nCoV). High body temperature, sore throat, cough, dyspnea, low energy, and weakness are common COVID-19 symptoms. In most people, the infection is mild, but in the elderly or those with co-morbidities it can proceed to pneumonia, respiratory distress, and multi-organ damage [1]. As of 16 August 2021, the WHO had received reports of 205,338,159 confirmed cases of COVID-19, with 4,333,094 deaths. A total of 4,428,168,759 vaccine doses had been provided by 13 August 2021 [2]. Many reviews on the pathophysiology, diagnosis and treatment of COVID-19 have also been published [3,4,5]. The global spread of SARS-CoV-2 has been impacting all human activities (health, social life, education, etc.), and economies [6]. It may continue for many months or perhaps years. Vaccines and antiviral drugs are important means of preventing and treating COVID-19. However, as of now only a few antiviral treatments are available for COVID-19 [5,6,7,8,9]. Even vaccinated people have been infected with SARS-CoV-2, millions of immunocompromised patients may not get full protection after vaccination, and the existing vaccines may not be effective against the new variants of SARS-CoV-2 [10]. Accordingly, the discovery of curative treatments is essential to fight COVID-19. Therefore, many strategies are being followed to discover more and better treatments for COVID-19, for example, the development of inhibitors of the key enzymes of SARS-CoV-2 like RNA-dependent RNA polymerase (favipiravir and remdesivir), papain-like protease (disulfiram), helicase (ivermectin), and 3-chymotrypsin-like protease (lopinavir and ritonavir) [11]. The therapeutic strategies to combat COVID-19 also revolve around the drug repurposing approach, i.e., identification of effective treatment from the pool of existing drugs [12]. Drug repurposing of FDA-approved chemical entities not only decreases the cost associated with drug discovery but also reduces the development time [13]. Several drugs with diverse mechanisms of action were clinically investigated to develop a potential treatment for COVID-19. Some of the repurposed drugs evaluated in clinical trials include antimalarial drugs (hydroxychloroquine and chloroquine), antiparasitic drugs (ivermectin), anti-inflammatory corticosteroids (dexamethasone and prednisolone), antibacterials (azithromycin), antivirals (lopinavir, ritonavir, and remdesivir), antihypertensives (losartan), immunomodulators, etc. [12]. Based on the current data, repurposed antiviral drugs inhibiting the RNA-dependent RNA polymerase (RdRp) enzyme appear to be the most successful and are therefore a promising drug target against COVID-19.

## 2. RNA-Dependent RNA-Polymerase (RdRp)

**Scheme 1:**
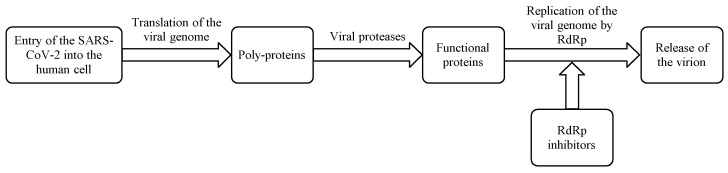
Mechanism of action of RdRp inhibitors.

RdRp is an attractive target for developing therapies for COVID-19 as it plays a crucial role in the replication of SARS-CoV-2 (Scheme 1) and is well conserved between coronaviruses (RNA viruses). The multi-domain proteins contain less than 500 units of amino acids in length. The protein looks like a human cupped right hand with three subfolded domains constituting thumb, palm, and fingers. There is no known equivalent of RdRp in humans and it therefore produces no off-target untoward effects, making RdRp a selective target to develop RdRp inhibitors. Further, the availability of biochemical assays accelerates the development of RdRp inhibitors [12,13,14,15,16]. Two RdRp inhibitors, remdesivir (anti-Ebola virus experimental drug) [8] and favipiravir (anti-influenza drug) [9] have already been approved for COVID-19 treatment (Table 1). Both these broad-spectrum antiviral drugs have been shown to reduce the progression of COVID-19 and associated clinical symptoms along with a substantial decrease in recovery time [17,18,19]. Remdesivir is administered intravenously but many pharmaceutical companies are developing its acceptable and convenient oral dosage forms. On the other hand, favipiravir is an orally active antiviral drug but it shows a poor pharmacokinetic profile. Hence, some other possible RdRp inhibitors are being considered for COVID-19 treatment, which includes molnupiravir, galidesivir, ribavirin, sofosbuvir, and tenofovir [14,15]. Recently molnupiravir, an orally active RdRp inhibitor with a favorable pharmacokinetic profile, has received considerable attention owing to its ability to inhibit SARs-COV-2 replication, its quick clearance of SARs-COV-2, and the accompanying reduction in viral load and fast recovery time [20]. Molnupiravir reaches quantifiable concentration in 0.5 h between 600–1600 mg. Administration of a single dose produces mean C_max_ values up to 13.2 ng/mL and shows median t_max_ between 0.25 and 0.75 h and a biological half-life (t_1/2__)_of 7 h. Its C_max_ and area under the plasma concentration versus time curve (AUC) increases in a dose-proportional manner with no accumulation following multiple doses suggesting that molnupiravir has no accumulative toxicity. Administration of molnupiravir in a fed state shows a slight decrease in the rate of absorption but no decrease in overall exposure. Furthermore, it exhibits fewer adverse reactions and good tolerability. Based on the pharmacokinetic profile of molnupiravir, it can be inferred that molnupiravir has a quick onset of action, a wide therapeutic window, and excellent tolerance with a good safety profile. These attributes make molnupiravir a very useful therapeutic molecule against COVID-19 [21].

## 3. Molnupiravir

**Figure 1:**
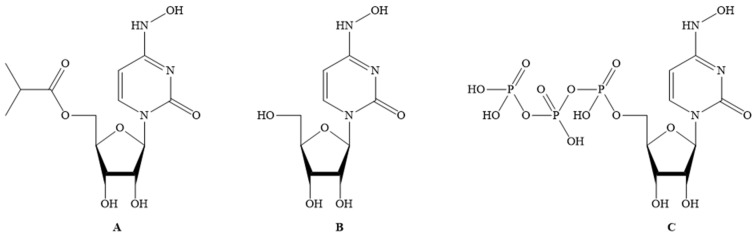
(**A**) Molnupiravir; (**B**) EIDD-1931; (**C**) EIDD-1931-triphopsphate.

Molnupiravir (MF: C_13_H_19_N_3_O_7_; MW: 329.31; CAS Registry Number: 2492423-29-5; Deleted CAS Registry Numbers: 2349386-89-4) is a pyrimidine ribonucleoside analog with a chemical name of ((2R,3S,4R,5R)-3,4-dihydroxy-5-(4-(hydroxyamino)-2-oxopyrimidin-1(2H)-yl) tetrahydrofuran-2-yl) methyl isobutyrate (Figure 1A). The other names of molnupiravir are EIDD-1931-isopropyl ester; EIDD-2801; MK-4482; uridine, 4-oxime, 5′-(2-methylpropanoate); and β-D-N4-hydroxycytidine-5′-isopropyl ester [21,22]. It is an orally active and directly acting antiviral interventional drug. Its efficacy and tolerability in COVID-19 patients has been investigated at doses of 200, 400, and 800 mg twice daily for five days.

### 3.1. Mechanism of Action

Molnupiravir acts by inhibiting RdRp of SARS-CoV-2 to induce RNA mutagenesis in two steps. Molnupiravir is converted to EIDD-1931 (CAS Registry Number: 3258-02-4; Deleted CAS Registry Numbers: 85373-26-8; MF: C_9_H_13_N_3_O_6_; MW: 259.22; Melting Point (Sci-finder): 169–172 °C) (Figure 1B) in the body, which on phosphorylation by host kinases provides the EIDD-1931-triphosphate (CAS Registry Number: 34973-27-8; MF: C_9_H_16_N_3_O_15_P_3_; MW: 499.16; Other names: 4-Oxime uridine-5′-triphosphate, EIDD-2061, N4-Hydroxy-CTP, and N4-Hydroxycytidine triphosphate) (Figure 1C). This triphosphate acts as an alternate/competitive substrate for the RdRp enzyme of SARS-CoV-2. Therefore, RdRp generates mutated RNA copies for SARS-CoV-2. This process causes the inhibition of the normal functions of RdRp [21]. Molnupiravir is a better electron donor than electron acceptor, and hence this reducing property can contribute to the antiviral activity as it affects the conditions required for viral infection.

### 3.2. Discovery and Development

Molnupiravir is an isopropyl prodrug of EIDD-1931 (Figure 1B). The other names of EIDD-1931 are *N*-Hydroxycytidine (NHC), and N4-Hydroxycytidine. EIDD-1931 demonstrated inhibitory effects on the replication of many viruses, including coronaviruses with promising safety profiles. However, EIDD-1931 also demonstrated poor bioavailability in animal models. After oral treatment, EIDD-1931 was rapidly metabolized in the enterocytes of nonhuman primates. The uptake and the distribution profile of EIDD-1931 in mice are available in the literature [23]. Molnupiravir was developed to overcome the poor bioavailability issue of EIDD-2801 [21,22,24].

Molnupiravir has been developed by scientists at Emory University (USA), with financial support from the USA government [25]. An agreement to develop molnupiravir as an oral treatment for non-hospitalized COVID-19 patients has also been signed between Emory University, Ridgeback Biotherapeutics, Wayne & Wendy Holman, and Merck [26]. Molnupiravir was originally intended to treat alphavirus infections. At the time of the beginning of the pandemic, it was in pre-clinical testing for seasonal influenza. After the spread of COVID-19, the molnupiravir development program moved to the treatment of COVID-19 [25].

#### 3.2.1. Pre-Clinical Studies

Molnupiravir demonstrated potent anti-influenza activity and good oral bioavailability in mice/ferrets/nonhuman primates [22]. This study also highlighted that molnupiravir shows antiviral effects because of its mutagenic property towards the influenza virus. The study further demonstrated a therapeutic window of >1713 (antiviral efficacy vs. cytotoxicity) and suggested further clinical studies of molnupiravir for influenza treatment. The pre-clinical study of molnupiravir in the animal model also showed its oral effectiveness against coronaviruses, including SARS-CoV and MERS-CoV [27]. This study supported the mutagenic property for SARS-CoV and MERS-CoV. The effectiveness of EIDD-1931 against remdesivir resistant virus was also demonstrated, which indicates that molnupiravir might be active against a broader range of viruses than remdesivir. Remdesivir inadequately controls SARS-CoV-2 transmission [28]. However, molnupiravir proved effective at reducing SARS-CoV-2 infection and blocking transmission in ferrets. Accordingly, molnupiravir has been suggested as a countermeasure to prevent community transmission of SARS-CoV-2 [28].

#### 3.2.2. Clinical Studies

The Phase 1 clinical data (pharmacokinetics, safety, and tolerability) of molnupiravir has recently been published [21]. The study was conducted in subjects over the age range of 19–60 years with a mean body mass index (BMI) of 24.4–25.4 kg/m^2^ in which male individuals were prominent. The pharmacokinetic profile of molnupiravir was evaluated in single and multiple-dose administrations in phase-1 randomized, double-blinded, and placebo-controlled clinical trials. In addition, the effect of food on drug pharmacokinetics was evaluated. The study revealed that molnupiravir absorbed well in plasma in the concentration range of 50–1600 mg in a dose-dependent manner. The rate of absorption of molnupiravir was quite low in the fed state. However, during exposure for longer durations, the absorption rate of both the fed and unfed states was similar. The main observed adverse event was headache. Molnupiravir did not exhibit any negative effects on vital functions and electrocardiogram data and had no clinically significant impact on hematological parameters. Molnupiravir has been considered quite safe at the dose levels of 50–1600 mg. The plasma t_1/2_ of molnupiravir is dose-dependent and ranges between 0.907 and 7.08 h. The tolerated dose was 50–800 mg BID in a multiple-ascending dose study and 50–1600 mg in a single dose study for 5.5 days. The effective dose for SARS-CoV-2 in humans was reported to be 200–800 mg. Accumulation of the drug was not seen in a multiple-dose study and molnupiravir has been proclaimed to be a promising pharmacological intervention for viral respiratory infections based on previous animal studies. Taken together, the study revealed that molnupiravir is well-tolerated and has dose-dependent pharmacokinetics when administered in healthy individuals at clinically relevant concentrations. It is expected that molnupiravir treatment may be 800 mg capsules two times a day for five days. A summary of the phase 1 clinical trials of molnupiravir is presented in Table 2.

Molnupiravir has completed its Phase 2 studies. However, the data of Phase 2 studies are not publicly available.

#### 3.2.3. Current Clinical Trials

Molnupiravir is undergoing seven clinical studies [www.clinicaltrials.gov] (accessed on 9 August 2021). A summary of these clinical studies is provided in Table 3.

**Scheme 2:**
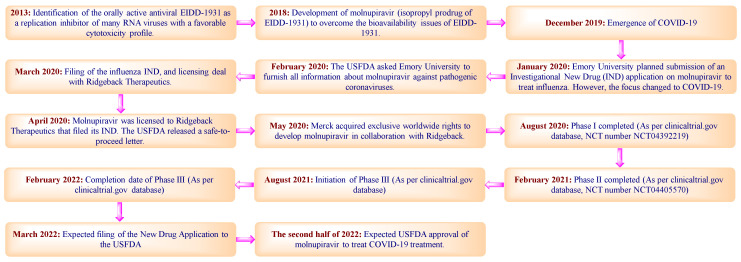
Summary of the development of molnupiravir.

The discovery and development timeline of molnupiravir has been summarized in Scheme 2.

## 4. Patent Searching

**Scheme 3:**
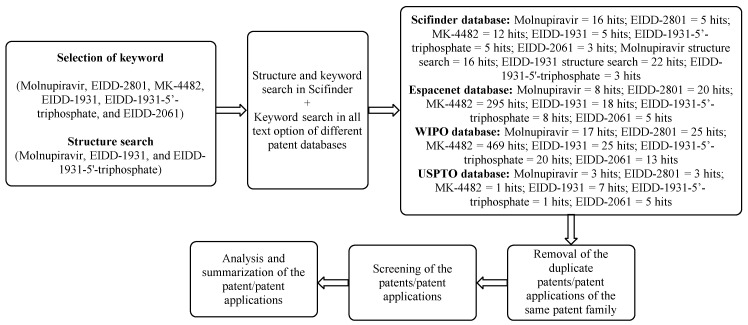
Methodology of patent searching.

The updated patent search was completed on 12 September 2021. The keywords molnupiravir, EIDD-2801, MK-4482, EIDD-1931, EIDD-1931-5′-triphosphate, and EIDD-2061 were selected. The patent searching was done in all text/field options of different databases (Espacenet, USPTO, WIPO, and Scifinder). The structure search of molnupiravir, EIDD-1931, and EIDD-1931-5′-triphosphate was also performed utilizing Scifinder. All the patents/applications related to molnupiravir, and EIDD-1931-5′-triphosphate were analyzed and included in this review. The patents/applications related to EIDD-1931 were also included if they were explicitly or implicitly related to virus infection or Emory University. The patent searching methodology is depicted in Scheme 3.

## 5. Patent Analysis

### 5.1. Compound Patent

**Scheme 4:**
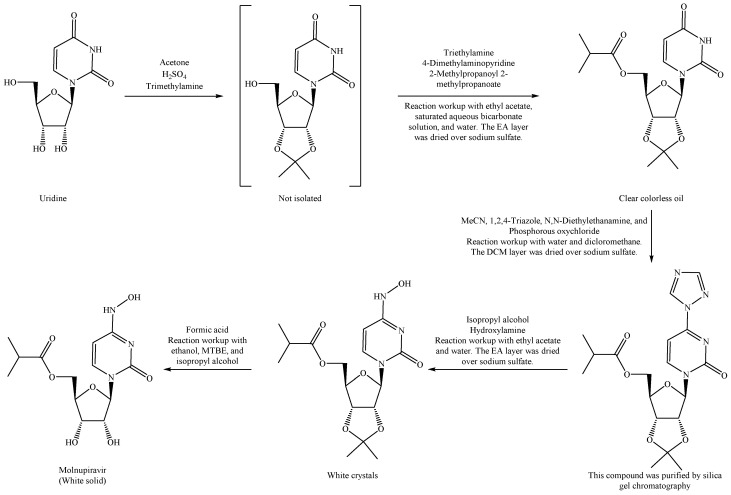
The summary of the process of preparing molnupiravir provided in US2020276219A1.

US2020276219A1 relates to N4-hydroxycytidine derivatives as antiviral agents [29]. This patent applies to molnupiravir and its salts specifically. It also covers a pharmaceutical composition comprising molnupiravir or its salts and a pharmaceutically acceptable excipient. It further claims to be a method of treating or preventing human coronaviruses, SARS coronavirus and MERS coronavirus infection, using an effective amount of molnupiravir or its salts. The synthesis of EIDD-1931, EIDD-1931-5′-triphosphate (EIDD-2061) and molnupiravir (EIDD-2801) are provided in examples 1, 2, and 10, respectively, of this patent. EIDD-1931 can be obtained by a few alternative synthetic routes. The shorter one-pot synthesis involves heating cytidine with pH-controlled hydroxylamine solution, but the product obtained is low yield and requires further purification. In the other method, uridine in dichloromethane is cooled to 0 °C under a nitrogen environment followed by the addition of 4-DMAP, imidazole, and finally 2,4,6-triisopropylbenzene-1-sulfonyl chloride (TBSCl). To the dark orange solution obtained after stirring the mixture for 18 h, hydroxylamine hydrochloride is added and then quenched with water. A dark orange oil so obtained is purified and treated with triethylamine trihydrofluoride. This mixture on workup produces EIDD-1931 with a 71% yield. Example 2 of the patent discloses the preparation of EIDD-2061. A mixture of cytidine triphosphate disodium salt and an aqueous solution of 2N hydroxylamine after adjusting pH to 5 is heated, cooled, and mixed with triethylammonium bicarbonate and then upon concentration by rotary evaporator produce the desired compound. The method of synthesis of molnupiravir (EIDD-2801) provided in example 10 in US2020276219A1 is depicted in Scheme 4. Briefly, uridine in acetone slurry is acidified with sulfuric acid and then stirred for 18 h at room temperature to protect the dihydroxy groups by forming acetonide. To the resulting mixture obtained after quenching with triethylamine (TEA), dimethylaminopyridine and TEA are added followed by gradual addition of 2-methylpropanoyl 2-methylpropanoate, and the reaction mixture is stirred until the reaction is over. The content is concentrated under reduced pressure and the residue is dissolved in ethyl acetate. The organic solution is washed twice each with a saturated solution of sodium bicarbonate, water, and brine. The organic layer upon concentration and drying gives a clear colorless oil of acetonide derivative. This oil is dissolved in acetonitrile followed by the addition of triazole and *N,N*-diethylethanamine. The solution obtained after stirring is further treated with phosphorous oxychloride and stirred under an argon environment. The organic layer is extracted, dried, concentrated, and purified using column chromatography. The solid product is dissolved in isopropyl alcohol and stirred with hydroxylamine at room temperature to replace the triazole ring with an -NH-OH group. At the completion of the reaction, the mixture is concentrated under a vacuum and suspended in ethyl acetate. Inorganic impurities are removed by washing with water and brine. The dried organic layer produced oil that forms a white solid upon standing. In the last step, molnupiravir is obtained as a white solid after an acidification reaction with formic acid to remove the acetonide ring to free the protected dihydroxy groups. This patent also revealed the low oral bioavailability of EIDD-1931 in cynomolgus monkeys due to its low solubility. However, when administered via intravenous injection, EIDD-1931 was widely distributed in the body. Accordingly, molnupiravir (isopropyl prodrug of EIDD-1931) was designed to facilitate the movement of EIDD-1931 from the gut wall to the circulating blood. The patent application also reported EIDD-1931-5′-triphosphate (EIDD-2061) as the active form of molnupiravir and EIDD-1931.

### 5.2. Polymorph Patent Application

CN112778387A discloses a molnupiravir crystal form A, characterized by its X-ray powder diffraction pattern, differential scanning calorimetry graph, and thermogravimetric analysis graph [30]. This application exemplifies the preparation of molnupiravir crystal form A using isopropanol (purity 99.8%, yield 91.7%), water (purity 99.5%, yield 83.3%), ethyl acetate (purity 99.6%, yield 94.5%), isopropyl acetate (purity 99.7%, yield 95.6%), dichloromethane (purity 99.7%, yield 86.4%), acetone (purity 99.6%, yield 79.2%), tetrahydrofuran (purity 99.7%, yield 89.5%), and methanol (purity 99.9%, yield 88.7%). The accelerated stability analysis of molnupiravir crystal form A (40 °C ± 2 °C and relative humidity 75% ± 5%) exhibited chemical stability, purity, and moisture content for two months under accelerated conditions.

### 5.3. Process Patent Applications

**Scheme 5:**
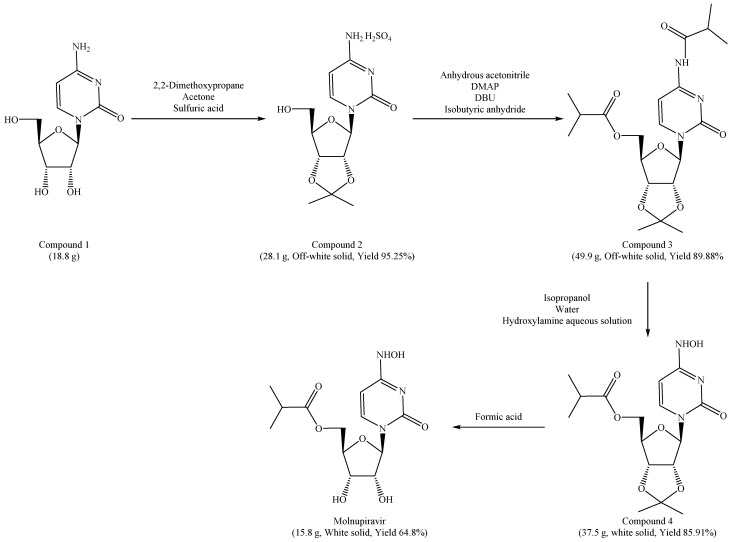
Summary of the process of preparing molnupiravir provided in CN112552288A.

CN112552288A discloses a process for preparing molnupiravir (Scheme 5) [31]. It states that the process for preparing molnupiravir mentioned in US2020276219A1 generates oily intermediates that are difficult to purify. This decreases the yield of molnupiravir and increases the cost of the process. The process in CN112552288A, it is claimed, is simple and produces molnupiravir with good crystallinity and yield (64.8%).

**Scheme 6:**
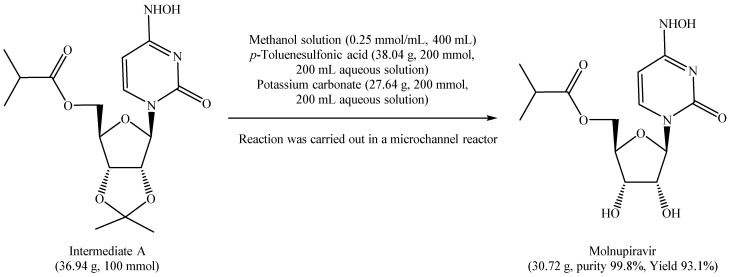
The summary of the process of preparing molnupiravir provided in CN112608357A.

CN112608357A relates to a process for preparing molnupiravir (Scheme 6) [32]. It states that the process of US2020276219A1 provides molnupiravir in poor yield, produces more waste, and is costly. This makes the process unsuitable for scale-up production. The process of CN112608357A utilizes a microchannel reaction technology to deprotect a solution of intermediate A and an acid solution in a microchannel reaction, and then neutralize it with an alkaline solution in a microchannel reactor to provide molnupiravir in a high yield. The claimed process is simple, low in cost, conducive to the synthesis of high-purity molnupiravir, and suitable for industrial production.

**Scheme 7:**
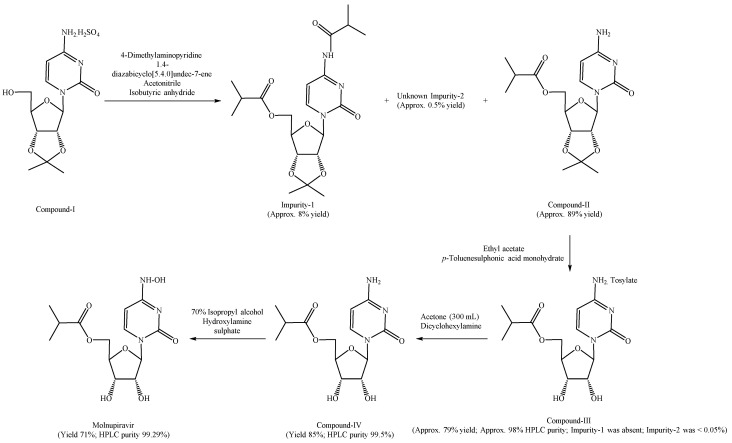
The summary of the process of preparing molnupiravir provided in IN202141018775A.

IN202141018775A discloses a new method of preparing molnupiravir (Scheme 7) [33]. It states that the process of US2020276219A1 uses uridine (expensive starting material) and provides molnupiravir in low yield. The application states the addition of a catalytic amount of 4-dimethylaminopyridine enhances the reaction (conversion of Compound-I to Compound-II). Compound-II is formed along with impurity-1 (about 8%) and an unknown impurity-2 (0.5%). The treatment of Compound-II with p-toluenesulphonic acid monohydrate yields tosylate salt (Compound-III), which was completely free from impurity-1, and impurity-2 was reduced to <0.05%. The conversion of a pure intermediate led to the production of molnupiravir in good yield.

### 5.4. Patents Related to EIDD-1931 and EIDD-2061

JP48000578A was the first patent that disclosed EIDD-1931 and its derivatives as antibacterial agents [34]. The first non-patent report [35] on EIDD-1931 was related to its mutagenic effects on bacterial cells. WO2017165489A1 discloses antiviral agents for treating Zika and dengue virus infections. It claims the use of EIDD-1931 for treating or preventing Zika virus (ZIKV) infection in a host [36]. US10874683B2 discloses N4-hydroxycytidines, including EIDD-1931 as antiviral agents. This patent provides the synthesis, pharmacokinetic parameters, and biological activity (Chikungunya Infection, Alphavirus Infections, Zika Infection, etc.) of EIDD-1931. This patent also mentions the structure of EIDD-2061 [37]. WO2016145142A1 relates to antiviral nucleotide and nucleoside therapeutic compositions. This application also provides the Venezuelan equine encephalitis virus (VEEV) assay data of EIDD-1931 [38]. AU2015370004B2 unveils the pharmacokinetic parameters and the biological activity (alphavirus infections and VEEV) of EIDD-1931. This patent also mentions the structure of EIDD-2061 [39]. US10149859B2 relates to sulfur-containing nucleosides (analogs of EIDD-1931) to treat viral infections (VEEV) [40]. US7049303B2 reported EIDD-1931 as JA29. The description of this patent mentioned in vitro toxicity data on the proliferation of human T-lymphocytes (CEM/O cells). This patent also mentioned that JA29 did not demonstrate any antiviral activity against HIV-1 infected CEM cells [41].

### 5.5. Miscellaneous Patent Applications

**Figure 2:**
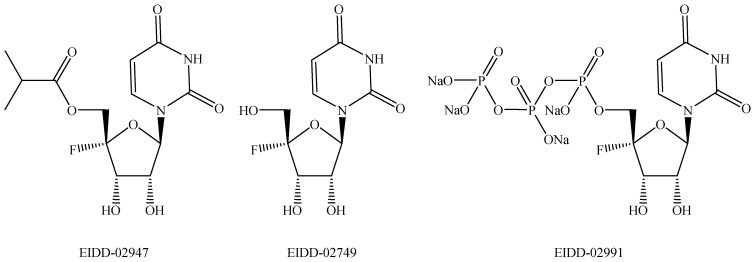
Chemical structures of EIDD-02749, EIDD-02991, and EIDD-02947.

WO2019173602A1 [42] and WO2021137913A2 [43] disclose halogen-containing nucleotide and nucleoside (analogs of molnupiravir) as antiviral against, for example, EIDD-02749, EIDD-02749-5′-triphosphate (EIDD-02991), and EIDD-02749-5′-Isobutyl ester (EIDD-02947) (Figure 2). These applications cover the use of claimed compounds for the treatment of positive-sense and negative-sense RNA viral infections by inhibiting the virally encoded RdRp. This application provides the activity of EIDD-02749 against numerous viruses, including coronavirus. It also mentions the pharmacokinetic, cytotoxicity, and stability data of EIDD-02749. These applications also provide the synthesis of molnupiravir in its specification.

IN202114023358 claims a transmucosal solid dosage (sublingual tablet) form of molnupiravir and remdesivir. This application states that the claimed sublingual tablet of molnupiravir has increased bioavailability as its first-pass metabolism is avoided. The application cites an example for remdesivir, but no example is mentioned for molnupiravir [44].

The patent data of the abovementioned patents/patent applications are provided in Table 4.

Our search also revealed some patent applications that disclose novel isolated antibodies [45], modified Trefoil Family Factor-2 (TFF2) polypeptide [46], 5-cyano derivatives of molnupiravir, and EIDD-1931 [47], thiazolo[4,5-*d*]pyrimidine based tetrahydrofuran derivatives [48], pyrrolo[2,1-*f*][1,2,4]triazine based tetrahydrofuran derivatives [49], peptidomimetics [50], imidazolidine derivatives [51], cell pathway inhibitors (rock inhibitors, Wnt inhibitors, glycogen synthesis kinase 3 (GSK-3) inhibitors, integrin inhibitors, IL-1 inhibitors, IL-6 inhibitors, and TGF beta inhibitors) [52], novel nucleosides [53], and hydrolyzable tannin (pentagalloyl glucose, chebulinic acid, chebulagic acid, pedunculagin, tellimagrandin I, tellimagrandin II, geraniin, corilagin, casuaricitin, and nupharin) for the treatment of COVID-19 alone or in combination with molnupiravir [54].

## 6. Conclusions

Molnupiravir is a promising oral treatment for COVID-19 that has been jointly developed by Emory University, Ridgeback Biotherapeutics, and Merck. This drug may be available to the public in the second quarter/half of 2022. The oral dosage form of molnupiravir will be more patient compliant than remdesivir injection. The postexposure prophylactic use of molnupiravir is also foreseeable. The compound patent of molnupiravir has been granted in the United States, and it may also be granted in Australia, Brazil, Canada, China, Europe, Britain, Japan, Korea, Philippines, and Singapore. The simple structure of molnupiravir allows for its easy synthesis. Therefore, many process-related patent applications are expected in the future that may help to reduce the cost of the marketed molnupiravir. Some more pharmaceutical patent applications of molnupiravir are also expected in the coming days. Molnupiravir has the potential to become a breakthrough therapy for COVID-19.

## 7. Expert Opinion

There is a substantial demand for a patient compliant, safe, tolerable, and an orally effective COVID-19 treatment for non-hospitalized patients. Molnupiravir meets these criteria. Molnupiravir has completed phase 1 and phase 2 clinical trials in about 15 months (Scheme 2). It is now in phase 3 of clinical trials. The fast development of molnupiravir as a COVID-19 treatment is because of the focused research of the scientists of Emory University, and the timely funding provided by the USA government and other agencies to antiviral research projects. Nucleoside-based antivirals are often potent, orally bioavailable, and can be converted to prodrugs on a needs basis, and resistance to nucleoside analogs is more difficult to develop than other antiviral classes and they are active against multiple RNA viruses [55]. Molnupiravir is also a pyrimidine-based ribonucleoside that has been developed starting from a natural ribonucleoside (Uridine) that was isolated from human plasma [26]. Molnupiravir also demonstrated activity against many RNA viruses. These features of molnupiravir also helped its rapid development as a treatment for COVID-19. The viricidal effect of molnupiravir is due to its mutagenic effects in the virus, wherein RdRp utilizes the active triphosphate form of molnupiravir (EIDD-2061, Figure 1C) as a substrate in place of cytidine/uridine triphosphate and generates mutated RNA copies [56]. A report states that active ribonucleosides, including molnupiravir, may also be mutagenic to the host [57]. However, another report mentions that molnupiravir has a sound safety profile [58]. Regulatory agencies have studied the toxicity data of molnupiravir and permitted its use in clinical trials. Accordingly, molnupiravir must be a non-mutagenic drug for the host.

Whenever a drug is discovered, the innovator files the patent application for its protection and to get a monopoly on the market for a specified time. Accordingly, molnupiravir patent applications have been filed. The patent search revealed important patents related to molnupiravir. The compound patent of molnupiravir has been granted by the USPTO [29] with an estimated expiry date of 7 December 2038. The USPTO provides up to 5 years’ extension to drug patents [59]. If molnupiravir is approved by the USFDA then the expiry date of the compound patent of molnupiravir [29] may also be extended beyond 7 December 2038. A similar type of patent extension is possible in other countries like Europe, Japan, and Australia. One patent application [30] claims molnupiravir crystal form A. However, the authors believe that molnupiravir crystal form A may also be obtained by following the process of preparing molnupiravir mentioned in its compound patent [29]. Accordingly, the polymorph patent application may face challenges from the Chinese patent office. The manufacturing process is related to the cost of the marketed medicine. A costly manufacturing process may increase the price of the medicine [8]. Therefore, the pharmaceutical industries always try to develop a cost-effective process for manufacturing a drug. The simple structure of molnupiravir makes its synthesis easy. Molnupiravir was originally synthesized from uridine (Scheme 4). Uridine is a costly starting material. Therefore, some new patented/non patented routes for the synthesis of molnupiravir utilizing cheaper starting materials (cytidine) have also been developed [31,32,33,60,61]. Our patent search also revealed IN202121014827A [62], IN202121005152A [63], and IN202141011933A [64], which also cover processes for preparing molnupiravir. However, at the time of revising this review, the complete specifications of these patent applications were not available. The authors believe that the complete specifications of these patent applications will be available at the Indian Patent Office website soon. One patent application related to the transmucosal solid dosage (sublingual tablet) form of molnupiravir has been filed. The authors trust that this application may be useful for an injectable drug, like remdesivir [8], and will not bring any advantageous effect to molnupiravir, which is already an orally active drug. The patent search also provided some patent applications related to new compounds, isolated antibodies, polypeptide, and hydrolyzable tannin that have been claimed to treat COVID-19 in combination with molnupiravir. However, these patent applications are silent about the clinical safety and efficacy of the claimed substances in combination with molnupiravir to treat COVID-19 [45,46,47,48,49,50,51,52,53,54]. Many types of patents are possible for a drug, including compound, polymorph, salt, isomer, intermediate, new indication, impurities, particle size, and dosage forms (tablet, capsule, drug combinations, etc.). The capsule may be the marketed form of molnupiravir. Therefore, patent applications related to capsules of molnupiravir may also be filed by many pharmaceutical companies. A combination of molnupiravir with favipiravir and other antivirals is also possible due to their synergistic effects for the treatment of new variants of SARS-CoV-2. At the time of completing this article, the compound, polymorph, and process patents have been identified. Accordingly, new patent applications related to salt, new indication, impurities, particle size, and dosage forms of molnupiravir are also foreseeable. The patent application filings of the inhalation dosage form of molnupiravir may also appear as such dosage forms provide better patient compliance and reduce toxicity. Molnupiravir is an isopropyl prodrug of EIDD-1931. Accordingly, other prodrugs of EIDD-1931 may also be assessed for COVID-19 and patented. Similarly, the clinically useful prodrugs of remdesivir are also anticipated [65].

On 9 June 2021, Merck announced its molnupiravir procurement agreement with the USA government [66]. According to this agreement, if molnupiravir receives emergency use authorization (EUA) from the USFDA for non-hospitalized COVID-19 patients, then Merck has to supply 1.7 million courses to the USA government. Merck is also in discussion with many collaborators/drug regulatory agencies outside the USA for global access to molnupiravir. Accordingly, Merck is investing in producing 10 million courses of molnupiravir by the end of December 2021. Further, one clinical Phase 3 study of molnupiravir (NCT04939428, 800 mg orally every 12 h for 5 days) (Table 3) is going to be completed on 3 April 2022. If this study is successful, then the sponsor can file its new drug application (NDA) with the USFDA in April 2022. Accordingly, we anticipate the EUA from USFDA for molnupiravir in the second half of 2022. If we trust the news on social media, then molnupiravir approval is closer in India and Australia. The USFDA has already approved injectable monoclonal antibodies [67] and intravenous remdesivir [8] as COVID-19 treatments. Therefore, oral treatment for non-hospitalized COVID-19 patients will be an advantage. Recently, Pfizer has also unveiled PF-07321332, an oral protease inhibitor that is in phase 1/2 clinical trials [68]. However, at present, molnupiravir looks a foreseeable treatment for COVID-19 that may be available to the public in the second half of 2022. Molnupiravir was developed to treat influenza [21]. It has also exhibited antiviral activity against a variety of viruses [27]. Therefore, the new indication of molnupiravir for other viral diseases is also imaginable. It is expected that commercial use of molnupiravir will be limited to non-hospitalized patients, whereas remdesivir may remain the preferred treatment for hospitalized patients.

## Figures and Tables

**Table 1:** Approved RdRp inhibitors in clinical practice.

Drug’s Name	Dosage Forms (Route/Dose)	Indications (Marketing Status)	Countries
Remdesivir (Veklury^®^)	Solution/Powder (Intravenous/200 mg loading dose, followed by 100 mg once daily for 5 to 10 days for adults)	COVID-19 patients of ≥12 years requiring hospitalization (Prescription)	Approved in >50 countries including USA, KSA, UAE and European Union
Favipiravir (FabiFlu^®^)	Film-coated tablet (Oral/1800 mg/dose twice a day on the first day; followed by 800 mg/dose twice a day for 7–10 days for adults)	COVID-19 (Prescription)	Approved in many countries, including China, India, Russia and Japan

**Table 2:** Summary of the phase 1 clinical trials of molnupiravir.

Type of Study	Total Participants	Dose	Pharmacokinetic Data
Interventional, phase 1, randomized, double-blind, placebo-controlled study	130	(i) A total of 64 subjects received a single oral dose of 50 to 1600 mg molnupiravir or placebo in the single-ascending-dose part.	Mean C_max_ up to 13.2 ng/mL and median t_max_ 0.25 and 0.75 h for doses in between 600–1600 mg. Excretion in urine (0.002%) for >800 mg dose. Geometric mean terminal elimination half-lives (t_1/2_) = 0.91–1.29 h postdose of drug up to 800 mg dose. Median t_1/2_ for 1200 and 1600 mg doses = 1.75 and 1.50 h
		(ii) A total of 55 subjects received twice-daily (BID) doses of 50 to 800 mg molnupiravir or placebo for 5.5 days in the multiple-ascending-dose part.	Median t_max_ in all dose cohorts of between 1.00 and 1.75 h postdose across both Days 1 and 6. At the 800-mg BID dose level, the mean t_1/2_ = 7.08 h AUCτ = 0.938–1.16; C_max_= 0.843–1.10 at all dose levels
		(iii) A total of 10 subjects received a single dose of 200 mg in the fed state followed by a single dose of 200 mg molnupiravir in the fasted state after a washout period of 14 days, or vice versa.	Mean C_max_—approximately 36% lower in the fed state compared to the fasted state AUC_inf_—similar for both fed and fasted states Mean t_1/2_ in fed and fasted treatments = 1.09 and 0.977 h
		(iv) One subject in the multiple-ascending-dose part received 800 mg molnupiravir BID for three days.	It was discontinued by the investigators.

**Table 3:** Interventional randomized clinical trials on molnupiravir (EIDD-2801, MK-4482) for the treatment of COVID-19.

Sponsor (Status)	Phase (Number Enrolled) (Interventions)	NCT Number (Other IDs)	Start Date (SD)/Completion Date (CD)/Last Update (LU)
Merck Sharp & Dohme Corp. (Active, not recruiting)	2/3 (304) (Molnupiravir/Placebo)	NCT04575584 (4482-001, 2020-003367-26, MK-4482-001, PHRR201210-003189, jRCT2031200404)	SD: 19 October 2020 CD: 10 August 2021 LU: 7 May 2021
Merck Sharp & Dohme Corp.(Recruiting)	2/3 (1850) (Molnupiravir/Placebo)	NCT04575597 (4482-002, 2020-003368-24, MK-4482-002, PHRR201209-003186, RCT2031210148)	SD: 19 October 2020 CD: 19 April 2022 LU: 5 August 2021
Merck Sharp & Dohme Corp. (Not yet recruiting)	3 (1332) (Molnupiravir/Placebo)	NCT04939428 (4482-013, 2021-000904-39, MK-4482-013)	SD: 16 August 2021 CD: 3 April 2022 LU: 4 August 2021
Ridgeback Biotherapeutics (Recruiting)	2 (96) (EIDD-2801/Placebo)	NCT04405739 (EIDD-2801-2004)	SD: 16 June 2020 CD: 8 December 2021 LU: 20 May 2021
Ridgeback Biotherapeutics (Completed)	1 (130) (EIDD-2801/Placebo)	NCT04392219 (EIDD-2801-1001, 2020-001407-17)	SD: 10 April 2020 CD: 11 August 2020 LU: 19 July 2021
Ridgeback Biotherapeutics (Completed)	2 (204) (EIDD-2801/Placebo)	NCT04405570 (EIDD-2801-2003)	SD: 16 June 2020 CD: 21 February 2021 LU: 23 February 2021
University of Liverpool (Recruiting)	1/2 (600) (EIDD-2801/Nitazoxanide/VIR-7832/VIR-7831/Placebo)	NCT04746183 (UoL001542)	SD: 3 July 2020 CD: 30 April 2022 LU: 20 May 2021

**Table 4:** Data of the important cited patents/applications.

Patent/Application Number (Priority Number and Priority Date) (Application Number and Application Date)	Assignee (Equivalent Publications on 12 September 2021)	International Patent Classification	Status (Database)
US2020276219A1 (US201762595907P, 7 December 2017; US2018064503W, 7 December 2018; US201816755779A, 7 December 2018; US201862626998P, 6 February 2018; US201862760434P, 13 November 2018) (US201816755779A, 7 December 2018)	Emory University (AU2018378832B2, BR112020010581A2, CA3082191A1, CN111372592A, EP3706762A1, GB2581936B, JP6804790B1, KR102248165B1, PH12020550607A1, SG11202004403QA, WO2019113462A1)	A61K31/7068; A61P31/12	Notice of Allowance Mailed as per USPTO (New patent number will be assigned soon)
CN112778387A (CN202110055824A, 15 January 2021) (CN202110055824A, 15 January 2021)	Hangzhou Kechao Biotechnology Co., Ltd. (None)	C07H1/00, C07H19/067	Request for examination filed (Espacenet)
CN112552288A (CN202110188342A, 19 February 2021) (CN202110188342A, 19 February 2021)	Nanjing Huaguan Biotechnology Co., Ltd. (None)	C07D405/04	Request for examination filed (Espacenet)
CN112608357A (CN202011515642A, 21 December 2020) (CN202011515642A, 21 December2020)	Hangzhou Kechao Biotechnology Co., Ltd. (None)	C07H1/00, C07H19/067, C12P17/16	Request for examination filed (Espacenet)
IN202141018775A (IN2021-41018775, 23 April 2021) (IN2021-41018775, 23 April 2021)	Divi’s Laboratories Ltd. (None)	C07H0001000000, A61P0031220000, C07D0277640000, C07D0207340000, C07D0317280000	Under Examination (Indian Patent Office)
JP48000578A (JP 1971-36614, 28 May 1971)	Mitsui Toatsu Chemicals Company Limited (None)	No information in Sci-finder	Expired (Based on the filing date mentioned on Sci-finder)
WO2017165489A1 (US201662312225P, 23 March 2016) (US2017023537W, 22 March 2017)	Emory University (None)	A61K31/4706, A61K31/635, A61P31/14	No entry in any national phase (Espacenet)
US10874683B2 (US201662306163P, 10 March 2016; US2017021759W, 10 March 2017; US201716083177A, 10 March 2017) (US201716083177A, 10 March 2017)	Emory University (WO2017156380A1)	A61K31/7068, A61P31/14, A01N43/04, A61K31/70, C07H19/067, C07H19/10, A61K9/00	Patented case (USPTO)
WO2016145142A1 (US201562130942P, 10 March 2015) (US2016021688W, 10 March 2016)	Emory University (None)	A61K31/7068, A61K31/7072, C07H19/10	No entry in any national phase (Espacenet)
AU2015370004B2 (US201462096915P, 26 December 2014; US2015066144W, 16 December 2015; US201562201140P, 5 August 2015) (AU2015370004A, 16 December 2015)	Emory University (BR112017013858A2, CA2972259A1, CN107427529A, EA201791460A1, EP3236972A1, IL252997A, JP2018500354A, KR20170123308A, SG11201705069YA, US2019022116A1, US2021060050A1, WO2016106050A1)	A61K31/7068; A61P31/18; A61P35/00; C07H19/067	Granted patent (Espacenet)
US10149859B2 (US201361876473P, 11 September 2013; US2014054930W, 10 September 2014; US201414917681A, 10 September 2014; US201461923317P, 3 January 2014; US201461986577P, 30 April 2014) (US201414917681A, 10 September 2014)	Emory University (EP3043803A1; JP6762873B2; TW201542581A; UY35732A; WO2015038596A1)	A61K31/7068, A61K45/06, A61K9/127, C07H19/10, C07H19/06	Patented case (USPTO)
US7049303B2 (GB0126701A, 7 November 2001; US20700502A, 30 July 2002) (US20700502A, 30 July 2002)	Medical Research Council (AU2002337388A1; EP1441744A2; JP2005507944A; WO03039450A2)	A01N43/90, A61K31/519, A61K31/52, A61K31/675, A61K31/7068, A61K31/7076, A61P31/14, C07H19/067, C07H19/16, C07H19/167, A61K31/70	Patent Expired Due to NonPayment of Maintenance Fees (USPTO)
WO2019173602A1 (US201862639725P, 7 March 2018) (US2019021168W, 7 March 2019)	Emory University (AU2019231725A1, BR112020018209A2, CA3093222A1, CN112074506A, EP3762372A1, GB2589205A, KR20200140274A, PH12020551404A1, SG11202008527WA)	A61K31/505, C07D239/10, C07D307/12, C07D405/04	National phase entry in AU, BR, CA, CN, EP, GB, KR, PH and SG (Espacenet)
WO2021137913A2 (US201962912165P, 8 October 2019) (US2020054857W, 8 October 2020)	Emory University (None)	C07H19/16;	No entry in any national phase (Espacenet)
IN202114023358 (IN202011038103, 3 September 2020) (IN202114023358, 25 May 2021)	Jubilant Generics Limited (None)	A61K0009200000, A61K0009000000, A61K0009480000, C07D0401120000, A61K0009160000	Under Examination (Indian Patent Office)
IN202121014827A (IN202121014827, 31 March 2021)	Fermenta Biotech Limited (None)	G06F021-62, C07C051-367, C07C209-08, H04L029-06, C07C227-02	Publication (Indian Patent Office)
IN202121005152A (IN202121005152, 6 February 2021)	Fermenta Biotech Limited (None)	C07C067-327, C07C037-20, C07D319-06, C12P041-00, C40B050-14	Publication (Indian Patent Office)
IN202141011933A (IN202141011933, 20 March 2021)	Optimus Drugs Private Limited (None)	C12N007-00, C01B021-02, C07K016-46, C07K014-755, B01J037-30	Publication (Indian Patent Office)

## Data Availability

Data available in a publicly accessible repository.

## References

[1] Rufaida M.T., Kedwai I., Ahsan F., Shamim A., Shariq M., Parveen S. (2021). A dossier on COVID-19 chronicle. J. Basic Clin. Physiol. Pharmacol..

[2] https://covid19.who.int.

[3] Couvreur P., Louvard D. (2021). COVID-19 and drugs: Pathophysiology and therapeutic approaches. Comptes Rendus Biol..

[4] Falzone L., Gattuso G., Tsatsakis A., Spandidos D.A., Libra M. (2021). Current and innovative methods for the diagnosis of COVID-19 infection (Review). Int. J. Mol. Med..

[5] Bestetti R.B., Furlan-Daniel R., Silva V.M.R. (2021). Pharmacological treatment of patients with mild to moderate COVID-19: A comprehensive review. Int. J. Environ. Res. Public Health.

[6] Das K., Pingali M.S., Paital B., Panda F., Pati S.G., Singh A., Varadwaj P.K., Samanta S.K. (2021). A detailed review of the outbreak of COVID-19. Front. Biosci. (Landmark Ed.).

[7] Chen B., Liu M., Huang C. (2021). Current diagnostic and therapeutic strategies for COVID-19. J. Pharm Analysis.

[8] Imran M., Alshrari A.S., Asdaq S.M.B. (2021). Trends in the development of remdesivir based inventions against COVID-19 and other disorders: A patent review. J. Infect. Public Health.

[9] Ghasemnejad-Berenji M., Pashapour S. (2021). Favipiravir and COVID-19: A Simplified Summary. Drug Res. (Stuttg).

[10] Christie A., Mbaeyi S.A., Walensky R.P. (2021). CDC Interim Recommendations for Fully Vaccinated People: An Important First Step. JAMA.

[11] Mei M., Tan X. (2021). Current Strategies of Antiviral Drug Discovery for COVID-19. Front. Mol. Biosci..

[12] Khan S.A., Al-Balushi K. (2021). Combating COVID-19: The role of drug repurposing and medicinal plants. J. Infect. Public Health.

[13] Pushpakom S., Iorio F., Eyers P.A., Escott K.J., Hopper S., Wells A., Doig A., Guilliams T., Latimer J., McNamee C. (2019). Drug repurposing: Progress.; challenges and recommendations. Nat. Rev. Drug Discov..

[14] Wang Y., Anirudhan V., Du R., Cui Q., Rong L. (2021). RNA-dependent RNA polymerase of SARS-CoV-2 as a therapeutic target. J. Med. Virol..

[15] Vicenti I., Zazzi M., Saladini F. (2021). SARS-CoV-2 RNA-dependent RNA polymerase as a therapeutic target for COVID-19. Expert Opin. Ther. Pat..

[16] Shu B., Gong P. (2016). Structural basis of viral RNA-dependent RNA polymerase catalysis and translocation. Proc. Natl. Acad. Sci. USA.

[17] Grein J., Ohmagari N., Shin D., Diaz G., Asperges E., Castagna A., Feldt T., Green G., Green M.L., Lescure F.X. (2020). Compassionate use of remdesivir for patients with severe Covid-19. N. Engl. J. Med..

[18] Wang Y., Zhang D., Du G., Du R., Zhao J., Jin Y., Fu S., Gao L., Cheng Z., Lu Q. (2020). Remdesivir in adults with severe COVID-19: A randomized, double-blind, placebo-controlled, multicentre trial. Lancet.

[19] Cai Q., Yang M., Liu D., Chen J., Shu D., Xia J., Liao X., Gu Y., Cai Q., Yang Y. (2020). Experimental treatment with favipiravir for COVID-19: An open-label control study. Engineering.

[20] Fischer W., Eron J., Holman W., Cohen M.S., Fang L., Szewczyk L.J., Sheahan T.P., Baric R., Mollan K.R., Wolfe C.R. (2021). Molnupiravir, an Oral Antiviral Treatment for COVID-19. medRxiv.

[21] Painter W.P., Holman W., Bush J.A., Almazedi F., Malik H., Eraut N.C.J.E., Morin M.J., Szewczyk L.J., Painter G.R. (2021). Human Safety, Tolerability, and Pharmacokinetics of Molnupiravir, a Novel Broad-Spectrum Oral Antiviral Agent with Activity Against SARS-CoV-2. Antimicrob. Agents Chemother.

[22] Toots M., Yoon J.J., Cox R.M., Hart M., Sticher Z.M., Makhsous N., Plesker R., Barrena A.H., Reddy P.G., Mitchell D.G. (2019). Characterization of orally efficacious influenza drug with high resistance barrier in ferrets and human airway epithelia. Sci. Transl. Med..

[23] From the American Association of Neurological Surgeons (AANS), American Society of Neuroradiology (ASNR), Cardiovascular and Interventional Radiology Society of Europe (CIRSE), Canadian Interventional Radiology Association (CIRA), Congress of Neurological Surgeons (CNS), European Society of Minimally Invasive Neurological Therapy (ESMINT), European Society of Neuroradiology (ESNR), European Stroke Organization (ESO), Society for Cardiovascular Angiography and Interventions (SCAI), Society of Interventional Radiology (SIR) (2018). Multisociety Consensus Quality Improvement Revised Consensus Statement for Endovascular Therapy of Acute Ischemic Stroke. Int. J. Stroke.

[24] Yoon J.J., Toots M., Lee S., Lee M.E., Ludeke B., Luczo J.M., Ganti K., Cox R.M., Sticher Z.M., Edpuganti V. (2018). Orally Efficacious Broad-Spectrum Ribonucleoside Analog Inhibitor of Influenza and Respiratory Syncytial Viruses. Antimicrob. Agents Chemother..

[25] Painter G.R., Natchus M.G., Cohen O., Holman W., Painter W.P. (2021). Developing a direct acting, orally available antiviral agent in a pandemic: The evolution of molnupiravir as a potential treatment for COVID-19. Curr. Opin. Virol..

[26] Wang Z., Yang L. (2020). Turning the Tide: Natural Products and Natural-Product-Inspired Chemicals as Potential Counters to SARS-CoV-2 Infection. Front. Pharmacol..

[27] Sheahan T.P., Sims A.C., Zhou S., Graham R.L., Pruijssers A.J., Agostini M.L., Leist S.R., Schäfer A., Dinnon K.H., Stevens L.J. (2020). An orally bioavailable broad-spectrum antiviral inhibits SARS-CoV-2 in human airway epithelial cell cultures and multiple coronaviruses in mice. Sci. Transl. Med..

[28] Cox R.M., Wolf J.D., Plemper R.K. (2020). Therapeutic MK-4482/EIDD-2801 Blocks SARS-CoV-2 Transmission in Ferrets. Res. Sq..

[29] Painter G.R., Bluemling G.R., Natchus M.R., Guthrie D.B. (2020). N4-hydroxycytidine and Derivatives and Anti-viral Uses Related Thereto. U.S. Patent.

[30] Xuchun Z., Yiping Z., Chenchen F. (2021). Molnupiravir Crystal form A and Preparation Method Thereof. CN Patent.

[31] Jian C., Changquan Y., Chenchen F. (2021). Preparation Method of 4-oxime-5′-(2-methylpropionyl) Uridine. CN Patent.

[32] Xuchun Z., Yiping Z., Chenchen F. (2021). Preparation Method of Antiviral Drug Molnbupiravir. CN Patent.

[33] Prasad M.K., Rao M.A. (2021). Process for the Preparation of Molnupiravir. IN Patent.

[34] Akira A., Kisaburo U., Tatsuro Y. (1973). Carbamoyloxycytidines. JP Patent.

[35] Sledziewska E., Janion C. (1980). Mutagenic Specificity of N4-hydroxycytidine. Mutat. Res..

[36] Schinazi R.F., Amblard F., Cox B.D., Bassit L., Zhou L., Gavegnano C. (2017). Antiviral Agents for Treating Zika and Dengue Virus Infections. WO Patent.

[37] Painter G.R., Guthrie D.B., Bluemling G.R., Natchus M.R. (2020). N4-hydroxycytidine and Derivatives and Anti-viral Uses Related Thereto. U.S. Patent.

[38] Liotta D.C., Painter G.R., Bluemling G.R., Rosa A.D.L. (2016). Nucleotide and Nucleoside Therapeutics Compositions and Uses Related Thereto. WO Patent.

[39] Bluemling G.R., Guthrie D.B., Natchus M.G., Painter G.R. (2021). N4-hydroxycytidine and Derivatives and Anti-viral Uses Related Thereto. AU Patent.

[40] Liotta D.C., Painter G.R., Bluemling G.R., Rosa A.D.L. (2018). Nucleotide and Nucleoside Therapeutic Compositions and Uses Related Thereto. U.S. Patent.

[41] Loakes D., Brown D.M., Negishi K., Moriyama K., Balzarini J. (2006). Inhibition of Viruses. U.S. Patent.

[42] Painter G.R., Perryman D., Bluemling G.R. (2019). 4′-halogen Containing Nucleotide and Nucleoside Therapeutic Compositions and Uses Related Thereto. WO Patent.

[43] Painter G.R., Perryman D., Bluemling G.R. (2021). 4′-halogen Containing Nucleotide and Nucleoside Therapeutic Compositions and Uses Related Thereto. WO Patent.

[44] Nandi I., Mukherjee T., Kumar D., Jain A., Soni P., Mistry G.N., Jaiswal N. (2021). Transmucosal Pharmaceutical Compositions of Antiviral Drugs. IN Patent.

[45] Lederman S. (2021). Anti-cd154 Antibodies and Uses Thereof. WO Patent.

[46] Lederman S., Daugherty B. (2021). Modified Tff2 Polypeptides. WO Patent.

[47] Li S., Yuan L. (2021). Substituted N4-hydroxycytidine Derivatives and Prodrugs Thereof for Use in Anti-novel Coronavirus Therapy. CN Patent.

[48] Qingyan B.O., Yuyan J.I.N., Tyagi G., Zhu Y. (2021). Method of Treating Virus Infection Using a tlr7 Agonist. WO Patent.

[49] Yuanchao X., Gengfu X., Yang H., Lei Z., Hualiang J., Jingshan S. (2021). Application of Nucleoside Analogue or Combination Preparation Containing Nucleoside Analogue in Resisting Virus. CN Patent.

[50] Schinazi R.F., Zandi K., Amblard F. (2021). Peptidomimetics for the Treatment of Coronavirus and Picornavirus Infections. U.S. Patent.

[51] Cho A., Link J.O., Xu J. (2020). 2-Imino-5-Oxo-Imidazolidine Inhibitors of HIV Protease. WO Patent.

[52] Peyman G.A. (2021). Method of Treating, Reducing, or Alleviating a Medical Condition in A Patient. U.S. Patent.

[53] Cihlar T., Osinusi A., Porter D.L. (2021). Methods for Treating Sars Cov-2 Infections. WO Patent.

[54] Chaudhuri R.K., Randhawa M. (2021). Multi-Targeted Compositions for Mitigating Acute Respiratory Distress Syndrome. U.S. Patent.

[55] Jordan P.C., Stevens S.K., Deval J. (2018). Nucleosides for the treatment of respiratory RNA virus infections. Antivir. Chem. Chemother..

[56] Kabinger F., Stiller C., Schmitzová J., Dienemann C., Kokic G., Hillen H.S., Höbartner C., Cramer P. (2021). Mechanism of molnupiravir-induced SARS-CoV-2 mutagenesis. Nat. Struct. Mol. Biol..

[57] Zhou S., Hill C.S., Sarkar S., Tse L.V., Woodburn B.M.D., Schinazi R.F., Sheahan T.P., Baric R.S., Heise M.T., Swanstrom R. (2021). β-d-N4-hydroxycytidine Inhibits SARS-CoV-2 Through Lethal Mutagenesis But Is Also Mutagenic To Mammalian Cells. J. Infect. Dis..

[58] Hiremath C.N. (2021). Abbreviated Profile of Drugs (APOD): Modeling drug safety profiles to prioritize investigational COVID-19 treatments. Heliyon.

[59] Imran M., Asdaq S.M.B., Khan S.A., Unnikrishnan M.D., Alamri A.S., Alsanie W.F., Alhomrani M., Mohzari Y., Alrashed A., AlMotairi M. (2021). Innovations and Patent Trends in the Development of USFDA Approved Protein Kinase Inhibitors in the Last Two Decades. Pharmaceuticals.

[60] Paymode D.J., Vasudevan N., Ahmad S., Kadam A.L., Cardoso F.S.P., Burns J.M., Cook D.W., Stringham R.W., Snead D.R. (2021). Toward a practical, two-step process for molnupiravir: Direct hydroxamination of cytidine followed by selective esterification. Org. Process. Res. Dev..

[61] Hughes D.T. (2021). Quest for a cure: Potential small-molecule treatments for COVID-19, Part 2. Org. Process. Res. Dev..

[62] Datla A., Nagre P., Tamore J., Prabhu M.S., Trivikram S., Kadam S.V., Degaonkar G.S., Muralidharan K., Rupraj N. (2021). Scalable Two Step Synthesis of Molnupiravir. IN Patent.

[63] Datla A., Nagre P., Tamore J., Prabhu M.S., Trivikram S. (2021). Chemo-Enzymatic Process for Synthesis of Molnupiravir. IN Patent.

[64] Reddy D.R.S., Reddy P.S., Rao G.V.G. (2021). Improved Process for Molnupiravir. IN Patent.

[65] Schooley R.T., Carlin A.F., Beadle J.R., Valiaeva N., Zhang X.Q., Clark A.E., McMillan R.E., Leibel S.L., McVicar R.N., Xie J. (2021). Rethinking remdesivir: Synthesis, antiviral activity and pharmacokinetics of oral lipid prodrugs. Antimicrob. Agents Chemother..

[66] https://www.merck.com/news/merck-announces-supply-agreement-with-u-s-government-for-molnupiravir-an-investigational-oral-antiviral-candidate-for-treatment-of-mild-to-moderate-covid-19/.

[67] (2021). An EUA for Bamlanivimab-A Monoclonal Antibody for COVID-19. JAMA.

[68] Pavan M., Bolcato G., Bassani D., Sturlese M., Moro S. (2021). Supervised Molecular Dynamics (SuMD) Insights into the mechanism of action of SARS-CoV-2 main protease inhibitor PF-07321332. J. Enzyme Inhib. Med. Chem..

